# Circ_0000620 acts as an oncogenic factor in gastric cancer through regulating MMP2 expression via sponging miR-671-5p

**DOI:** 10.1186/s40709-021-00154-5

**Published:** 2021-12-31

**Authors:** Junyu Ren, Guoqing Pan, Jun Yang, Ning Xu, Qiong Zhang, Wenliang Li

**Affiliations:** 1 Department of Oncology, First Affiliated Hospital of Kunming Medical University, No. 295, Xichang Road, Wuhua District, 650032 Kunming, China; 2 Department of Pathology, First Affiliated Hospital of Kunming Medical University, Kunming, China; 3 Department of Anesthesiology, First Affiliated Hospital of Kunming Medical University, Kunming, China

**Keywords:** Gastric cancer, Angiogenesis, Metastasis, circ_0000620, miR-671-5p, MMP2

## Abstract

**Background:**

Gastric cancer (GC) is one of the most common cancers in the digestive system. Circular RNAs (circRNAs) have been found to function as important regulators in the pathogenesis of GC. This study focused on the biological role and molecular mechanism of circ_0000620 in GC progression.

**Methods:**

The expression levels of circ_0000620, microRNA-671-5p (miR-671-5p) and Matrix MetalloProteinase 2 (MMP2) were measured by quantitative real-time polymerase chain reaction (qRT-PCR), immunohistochemistry (IHC) assay or western blot. The stability of circ_0000620 was confirmed by Ribonuclease R (RNase R) assay. The protein levels were determined by western blot assay. Cell viability, colony formation, cell migratory ability, cell invasive ability and tube formation capacity were respectively examined by CCK-8 assay, colony formation assay, wound healing assay, transwell invasion assay and tube formation assay. The interaction between miR-671-5p and circ_0000620 or MMP2 was validated by dual-luciferase reporter assay, RNA immunoprecipitation (RIP) assay and RNA pull-down assay. The role of circ_0000620 in GC *undefined* was explored by xenograft tumor assay.

**Results:**

Circ_0000620 was conspicuously upregulated in GC tissues and cells. Circ_0000620 knockdown reduced cell viability, colony formation, migration, invasion and tube formation capacity of GC cells in vitro. Furthermore, MMP2 was upregulated in GC and MMP2 overexpression reversed the anti-tumor response of circ_0000620 knockdown in GC progression. Moreover, circ_0000620 directly interacted with miR-671-5p and circ_0000620 downregulation regulated malignant behaviors of GC cells by upregulating miR-671-5p. In addition, silencing of circ_0000620 inhibited tumor growth in vivo.

**Conclusions:**

Circ_0000620 knockdown inhibited the malignant development of GC partly through modulating the miR-671-5p/MMP2 axis.

**Supplementary Information:**

The online version contains supplementary material available at 10.1186/s40709-021-00154-5.

## Background

Gastric cancer (GC) is one of the most common malignancies and accounts for cancer-induced deaths worldwide, especially in China [1–3]. Despite GC treatment has been improved, the overall prognosis is still poor due to tumor metastasis [4–6]. GC progression is associated with various pathological events, including angiogenesis, proliferation, migration and invasion [7]. In addition, anti-angiogenic drugs (e.g. sorafenib and regorafenib) have been approved for the treatment of GC [8, 9]. Angiogenesis is a crucial for wound healing, tissue growth/regeneration, and cancer progression [10]. Tube formation in endothelial cells (ECs) is the prerequisite for tumor angiogenesis [11, 12]. Thus, it is necessary to investigate the molecular mechanism related with tumor metastasis and angiogenesis in GC.

CircRNAs are special endogenous RNAs containing covalently closed-loop structures [13]. CircRNAs exhibit many molecular functions, such as microRNA (miRNA) sponges or protein scaffolding in the progression of human malignancies [14, 15]. Additionally, circRNAs can affect gene expression via the miRNA sponging effect in GC [16]. For example, circ_0081146 facilitated the development of GC via sponging miR-144 to regulate the level of HMGB1 [17]. Circ-ITCH suppressed the metastasis of GC via regulating miR-199a-5p/Klotho axis [18]. The recent study indicated that many circRNAs were dysregulated in GC cells [19]. Three upregulated circRNAs (circ_0000620, circ_0000847, circ_0000567) were selected as the candidate subjects. The preliminary experiment demonstrated that circ_0000620 was upregulated with the most significant change in GC samples (Additional file 1; Fig. S1). Nevertheless, the biological function and molecular mechanism of circ_0000620 in GC remain to be explored.

MicroRNAs (miRNAs) is a class of small non-coding RNAs that can negatively regulate the levels of target genes [20]. Increasing miRNAs have been reported to be implicated in tumor initiation, progression and angiogenesis [21, 22]. MiR-671-5p was verified to regulate the carcinogenesis of diverse cancers, such as prostate cancer [23] and osteosarcoma [24]. Moreover, Qiu et al. suggested that miR-671-5p inhibited GC cell proliferation and promoted cell apoptosis by targeting URGCP [25]. However, the potential interaction between miR-671-5p and circ_0000620 in GC is still unknown.

Matrix MetalloProteinase 2 (MMP2, gelatinase A) is one of secreted zinc-dependent endopeptidases with vital roles in cell survival, migration, invasion and angiogenesis [26]. For instance, MMP2 downregulation weakened the migratory and invasive abilities of breast cancer cells [27]. MMP2 knockdown suppressed angiogenesis of human dermal microvascular endothelial cells-1 (HMEC-1) in lung cancer cells [28]. Some studies revealed that MMP2 served as an oncogene in GC [29]. Moreover, MMP2 have been found to be related to angiogenesis of GC cells [30, 31]. It is unclear whether MMP2 can be a target of miR-671-5p and circ_0000620 can regulate MMP2 expression via miR-671-5p .

Herein, we investigated the expression and function of circ_0000620 in GC development. The potential mechanism of circ_0000620 with miR-671-5p and MMP2 was also elucidated in the present study, aiming to promote the molecular understanding of circ_0000620 in GC progression.

## Methods

### Clinical samples and cell culture

GC patients (n = 44, male : female = 1 : 1, 40-70 years old) from First Affiliated Hospital of Kunming Medical University between January 2016 and December 2016 were recruited in this study. All patients did not receive any treatment before surgery. GC tissues and adjacent normal tissues were collected from these patients during radical gastrectomy. The partial samples were snap-frozen in liquid-nitrogen and then stored at −80 °C. In addition, other samples were fixed with formalin and embedded with paraffin for immunohistochemistry (IHC) assay. This study was performed with the acquisition of written informed consents from patients and the approval of Ethics Committee from First Affiliated Hospital of Kunming Medical University.

Human GC cell lines (HGC27 and AGS) were obtained from China Center for Type Culture Collection (Wuhan, China). HGC27 is from undifferentiated GC cells with epithelial morphology, and AGS is from gastric adenocarcinoma cells with epithelial morphology. Human umbilical vein endothelial cells (HUVECs) were purchased from American Type Culture Collection (ATCC, Manassas, VA, USA). Human gastric epithelial cells (GES-1) were purchased from Shanghai Institute of Biochemistry and Cell Biology, Chinese Academy of Sciences (Shanghai, China). HUVECs were maintained in Endothelial Cell Growth Medium (Sigma-Aldrich, St. Louis, MO, USA). GES-1, HGC27 and AGS cells were maintained in RPMI-1640 medium (Thermo Fisher Scientific, Rockford, IL, USA) supplemented with 10% fetal bovine serum (FBS, Thermo Fisher Scientific, Rockford, IL, USA). All cells were cultured in a humidified incubator containing 5% CO_2_ at 37 °C.

### Cell transfection

Short hairpin RNA (shRNA) vector targeting circ_0000620 (sh-circ_0000620: AATTCAAAAAGTGATGAAGAATGATATCCTTCTCGAGAAGGATATCTTCTTCATCAC), shRNA negative control (sh-NC: CCGGCAACAAGATGAAGAGCACCAACTCGAGTTGGTGCTCTTCATCTTGTTGTTTTTG), miR-671-5p mimic or inhibitor (miR-671-5p or anti-miR-671-5p), mimic or inhibitor control (miR-NC or anti-miR-NC), MMP2 overexpression vector (MMP2) and the pcDNA control vector were all obtained from GenePharma Co., ltd (Shanghai, China). The oligonucleotides or plasmids were transfected into HGC27 and AGS cells using Lipofectamine 3000 reagent (Thermo Fisher Scientific, Rockford, IL, USA) following manufacturer’s instruction. Transfection concentrations were 40 nM shRNA vector, 40 nM mimic, 20 nM inhibitor, 2 µg MMP2 or pcDNA vector, respectively.

### Quantitative real-time polymerase chain reaction (qRT-PCR)

Total RNAs were isolated from tissues and cells using Trizol reagent (Thermo Fisher Scientific, Rockford, IL, USA). Then, mature miR-671-5p was quantified using TaqMan MicroRNA Assays (Thermo Fisher Scientific, Rockford, IL, USA) with U6 as the internal control. Reverse transcription by M-MLV Reverse Transcriptase (Thermo Fisher Scientific, Rockford, IL, USA) and PCR reaction by SYBR™ Green Master Mix (Thermo Fisher Scientific, Rockford, IL, USA) were used for the determination of circ_0000620, AAGAB and MMP2. The reaction protocols were listed as below: pre-denaturation at 95˚C for 30 s, 40 cycles of denaturation at 95˚C for 5 s and annealing at 60˚C for 30 s. Glyceraldehyde-3-phosphate dehydrogenase (GAPDH) functioned as the house-keeping gene to normalize circ_0000620, AAGAB and MMP2 levels. The primer sequences were as follows: 5΄-CTGAATGCCAATGTGTGGTC-3΄ (forward) and 5΄-CTATCAAGGCCCGATTTTTG-3΄ (reverse) for circ_0000620; 5΄-GGCAAAAGCATGGTTACCTGAGG-3΄ (forward) and 5΄-CTCAGGCAACTCCTCTGGACTA-3΄ (reverse) for AAGAB; 5΄-GCCGAGAGGAAGCCCTGGAG-3΄ (forward) and 5΄-CTCAACTGGTGTCGTGGA-3΄ (reverse) for miR-671-5p; 5΄-GATGGCACCCATTTACACCTAC-3΄ (forward) and 5΄-GTCCTTGAAGAAGAAGATCTC-3΄ (reverse) for MMP2; 5΄-GTCAGTGGTGGACCTGACCT-3΄ (forward) and 5΄-CCCTGTTGCTGTAGCCAAAT-3΄ (reverse) for GAPDH; and 5΄-CTCGCTTCGGCAGCACA-3΄ (forward) and 5΄-AACGCTTCACGAATTTGCGT-3΄ (reverse) for U6.

### Ribonuclease R (RNase R) assay

The isolated total RNA samples from HGC27 and AGS cells were exposed to 3 U µg^−1^ RNase R (Epicentre, Madison, WI, USA) for 15 min at 37℃. The expression levels of circ_0000620 and linear AAGAB were determined by qRT-PCR assay. RNA without incubation of RNase R was used as the negative control group (Mock).

### Cell Counting Kit-8 (CCK-8) assay

Cell viability was tested using a CCK-8 assay kit (Dojindo Molecular Technologies, Rockville, MD, USA) according to the instructions of manufacturer. Briefly, the transfected HGC27 and AGS cells were seeded into 96-well plates and cultured for 24 h in fresh medium. Then, cells were incubated with CCK-8 solution (10 µL per well) for 3 h and cell absorbance was measured at 450 nm.

### Colony formation assay

1000 cells per well were plated into 6-well plates and cultured for 14 days. Subsequently, cells were fixed with 4% formaldehyde for 15 min and stained with 0.1% crystal violet (Sigma-Aldrich, St. Louis, MO, USA) for 10 min. The colonies (containing more than 50 cells) were counted under an optical microscope.

### Wound healing assay

Cell migration ability was analyzed by wound healing assay. HGC27 and AGS cells were seeded in 6-well plates and transfected for 24 h, followed by producing two scratches via a sterile pipette tip (200 µL). Whereafter, cells were gently washed using PBS twice and maintained in serum-free medium (SFM) for 24 h. Images of these scratches were captured at 0 and 24 h under a microscope with 40 × magnification. The migration rate (%) was calculated by the formula: (wound width_0 h_ - wound width_24 h_)/wound width_0 h_.

### Transwell invasion assay

The invasive potential was tested using a matrigel-precoated Transwell Boyden Chamber (Costar, Lowell, MA, USA) containing 8 μm pore size membranes. Cells resuspended in SFM were plated on the upper chamber, and medium with 10% FBS was added into the lower compartment. After 24 h of incubation, cells on the upper surface of membranes were removed. Cells attached to the lower surface of membranes were fixed with methanol for 20 min and stained with 0.1% crystal violet solution (Sigma-Aldrich, St. Louis, MO, USA) for 20 min. The invaded cell images were acquired and counted under the inverted microscope (Olympus, Tokyo, Japan).

### Collection of GC cells-conditioned medium (CM)

HGC27 and AGS cells were plated into the 6 well plates and then transfected with or without corresponding miRNA mimic, siRNAs, or plasmids, alone or in combination. At 36 h after transfection, the medium was removed and cells were maintained in SFM for 12 h. Next, the collected CM was centrifuged at 3000 rpm for 10 min to remove cells and at 12,000 rpm for 10 min to eliminate cell debris. Finally, CM was stored in aliquots at −80 °C.

### Tube formation assay

Tube formation ability of HUVECs was assessed by In vitro Angiogenesis Assay Kit (Millipore, Bedford, MA, USA) following the protocols of manufacturer. Briefly, HUVECs were seeded into 96-well plates precoated with ECMatrix^TM^ and cultured in SFM or CM for 12 h. Then, the average values of branch points were counted in 10 random views of fields under a phase contrast microscope (CK40; Olympus, Tokyo, Japan).

### Western blot assay

The total proteins were extracted using RIPA Lysis and Extraction Buffer (Thermo Fisher Scientific, Rockford, IL, USA) containing protease inhibitor cocktail (Roche Diagnostics, Mannheim, Germany). Then, protein concentration was measured using Bio-Rad Protein Assay (Bio-Rad, Hercules, CA, USA). Next, 30 µg proteins were separated by SDS-PAGE and electrotransferred onto nitrocellulose membranes (Millipore, Billerica, MA, USA). Subsequently, the membranes were blocked with 5% skim milk followed by incubation with the primary antibodies and the secondary antibody. The antibody information was shown as below: anti-MMP2 (1:1000; ab97779; Abcam, Cambridge, MA, USA), E-cadherin (E-cad, ab133597; 1:2000; Abcam, Cambridge, MA, USA), N-cadherin (N-cad, ab207608; 1:1000; Abcam, Cambridge, MA, USA), and anti-GAPDH (1:5000; ab9485; Abcam, Cambridge, MA, USA) and horseradish peroxidase (HRP)-labeled goat anti-rabbit secondary antibody (1:5000; ab205718; Abcam, Cambridge, MA, USA). Finally, protein signals were detected using Clarity Western ECL Substrate (Bio-Rad, Hercules, CA, USA) and the signal intensity was estimated using Quantity One software Version 4.1.1 (Bio-Rad, Hercules, CA, USA) via gray analysis.

### IHC assay

MMP2 protein expression was determined using western blot, with nine pairs of tissues as the experimental samples. The formalin-fixed and paraffin-embedded tissues were suffered from the sequential treatment of antigen retrieval, endogenous peroxidase blockage, nonspecific signal blocking. Then, tissue sections were incubated with anti-MMP2 primary antibody and HRP-conjugated secondary antibody. Then, tissue slices were stained with 3,3’-diaminobezidin (DAB) substrate and counterstained with hematoxylin. The stained tissues were imaged using an inverted microscope (Nikon E-800 M; Nikon Corporation, Japan).

### Subcellular fractionation assay

RNA was obtained from the cytoplasm and nucleus of HGC27 and AGS cells using Cytoplasmic & Nuclear RNA Purification Kit based on the guidance of manufacturer (Norgen Biotek, Thorold, ON, Canada). 18 S rRNA and U6 were used as internal controls for cytoplasm and nucleus.

### Dual-luciferase reporter assay

The online circinteractome (https://circinteractome.nia.nih.gov/), circbank (http://www.circbank.cn/index.html) and starbase (http://starbase.sysu.edu.cn) were used for predicting the binding sites between targets. The sequence of circ_0000620 or MMP2 3΄ untranslated region (3΄UTR) containing predicted or mutant miR-671-5p binding site was constructed into psiCHECK-2 luciferase vector by Hanbio Biotechnology Co., ltd. (Shanghai, China). The novel plasmids were named as circ_0000620 WT, MMP2 3΄UTR-1 WT and MMP2 3΄UTR-2 WT, circ_0000620 MUT, MMP2 3΄UTR-1 MUT and MMP2 3΄UTR-2 MUT. Then, HGC27 and AGS cells in 24-well plates were co-transfected with 100 ng reporter plasmids and 30 nM miR-671-5p or miR-NC using Lipofectamine 3000 reagent. Forty-eight hours later, luciferase activities were detected using a dual luciferase reporter assay system (Promega, Madison, WI, USA).

### RNA immunoprecipitation (RIP) assay

After transfection for 48 h, HGC27 and AGS cells were lysed using RIP lysis buffer (Bio-Rad, Hercules, CA, USA). Subsequently, cell lysates were incubated with Magnetic beads (Bio-Rad, Hercules, CA, USA) containing Argonaute 2 antibody (Ago2; Bio-Rad, Hercules, CA, USA) or Immunoglobulin G antibody (IgG; Bio-Rad, Hercules, CA, USA) for 3 h at 4℃. The RNA levels of circ_0000620, miR-671-5p and MMP2 were detected by qRT-PCR after RNA extraction using TRIzol reagent (Takara, Dalian, China).

### RNA pull-down assay

Biotin-labeled wild type miR-671-5p (WT-bio-miR-671-5p), biotin-labeled mutant miR-671-5p (MUT-bio-miR-671-5p) and control probe (bio-miR-NC) were bought from Thermo Fisher Scientific (Rockford, IL, USA). Briefly, HGC27 and AGS cells were harvested and re-suspended in RIPA lysis buffer. Cell lysates and WT-bio-miR-671-5p, MUT-bio-miR-671-5p or bio-miR-NC were incubated for 1 h, followed by addition of streptavidin agarose beads for 1 h. Finally, qRT-PCR was used for measuring the enrichment of circ_0000620 and MMP2.

### Mouse xenograft assay

A mouse xenograft model was established by subcutaneous injection with sh-circ_0000620 or sh-NC-transfected HGC27 cells into nude mice (five for each group). Tumor volume (length × width^2^/2) was measured once a week. The mice were sacrificed after 5 weeks, then the tumor tissues were resected and weighed. All animal experiments were carried out in line with the protocols approved by the Institutional Animal Care and Use Committee of First Affiliated Hospital of Kunming Medical University.

### Statistical analysis

All experiments were repeated for three times with the data exhibition as the mean ± standard deviation. The normality of data distribution was analyzed using the Kolmogorov-Smirnov test, and the homogeneity of variances was assessed using the Levene test. Data were analyzed by SPSS 22.0 (SPSS Inc., Chicago, IL, USA). Difference analysis was conducted through Student’s *t*-test and one-way ANOVA followed by Tukey’s test, with *p* level set at < 0.05 (as a statistically significant difference). The linear correlation between circ_0000620 and MMP2 was analyzed by Pearson’s correlation coefficient in clinical tissues. Survival analysis was performed by log-rank test. All figures were plotted using GraphPad Prism software (GraphPad Inc., La Jolla, CA, USA).

## Results

### Circ_0000620 was upregulated in GC tissues and cells

**Fig. 1:**
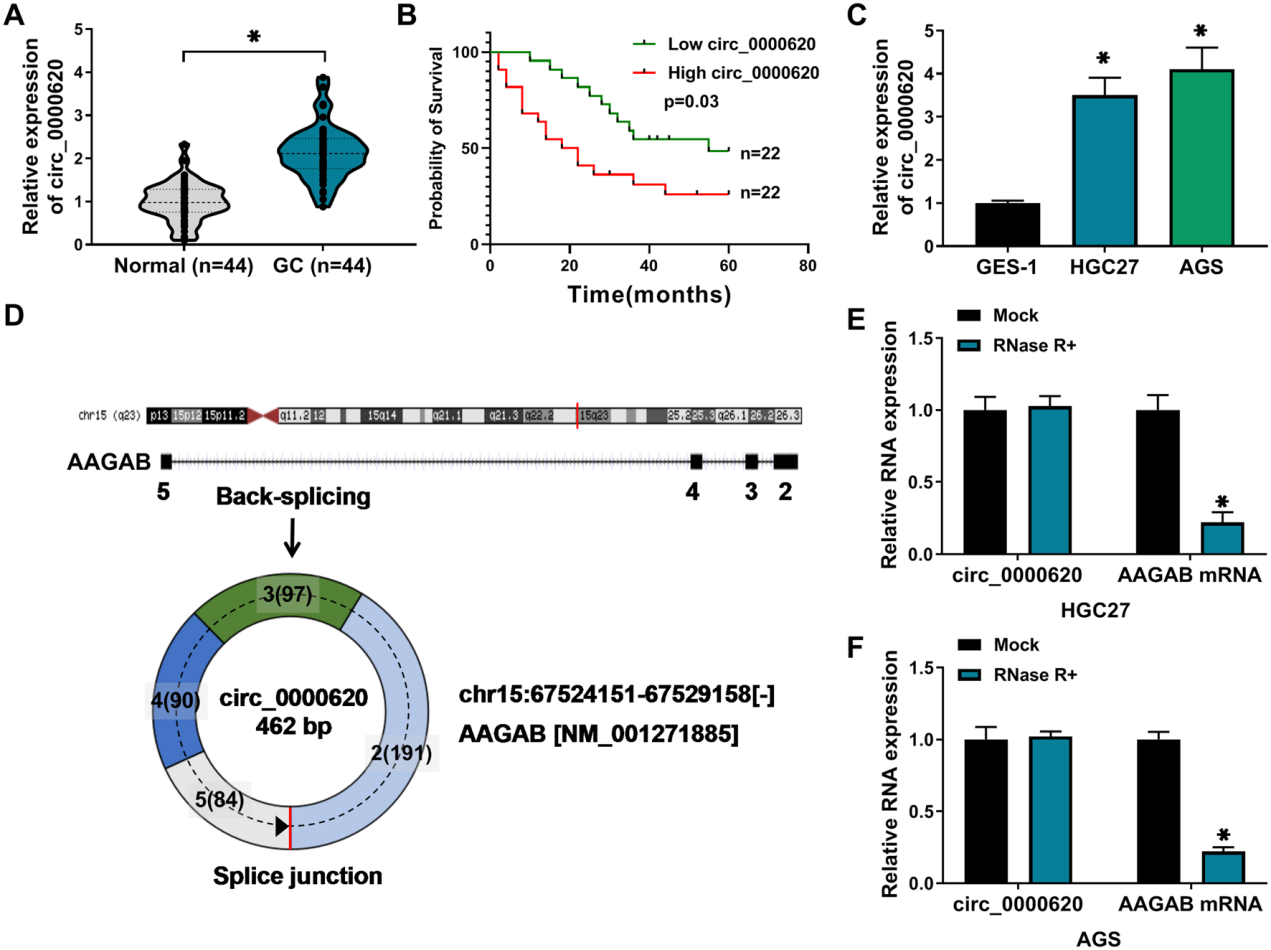
Circ_0000620 abundance was enhanced in GC tissues and cells. **A** The expression of circ_0000620 was quantified by qRT-PCR in 44 pairs of tumor tissues and matched non-tumor tissues. **B** Survival analysis was conducted by Log-rank test in GC patients with high or low circ_0000620 level. **C** The circ_0000620 level was determined using qRT-PCR in GES-1, HGC27 and AGS cells. **D** The schematic diagram of circ_0000620 formation. **E** **F** RNase R assay was used to evaluate the stability of circ_0000620 and linear mRNA AAGAB. Three repetitions were performed in each experiment, with three parallels every time. *p < 0.05

To explore the role of circ_0000620 in GC, circ_0000620 expression was firstly detected in GC tissues and cells. The qRT-PCR assay showed that circ_0000620 was significantly upregulated in GC tissues (n = 44) relative to adjacent normal tissues (n = 44) (Fig. 1A). Forty four patients were divided into high circ_0000620 expression group (n = 22) and low expression group (n = 22) according to the median value. The survival analysis revealed that survival rate was decreased in GC patients with high circ_0000620 expression compared to those patients with low circ_0000620 expression, suggesting that circ_0000620 could predict poor prognosis for GC patients (Fig. 1B). Also, circ_0000620 level was increased in GC cells (HGC27 and AGS) compared with GES-1 cells (Fig. 1C). Circ_0000620 is derived from the back-splicing of exon2-exon5 of alpha and gamma adaptin binding protein (AAGAB) gene, and the schematic diagram of circ_0000620 was shown in Fig. 1D. RNase R assay presented that RNase R reduced the expression of linear AAGAB mRNA but did not affect the expression of circ_0000620 compared to Mock group (Fig. 1E, F), implying that circ_0000620 was highly stable in GC cells. Thus, circ_0000620 was an upregulated circRNA in GC.

### Effects of circ_0000620 knockdown on the proliferation, tube formation and invasion of GC cells

**Fig. 2:**
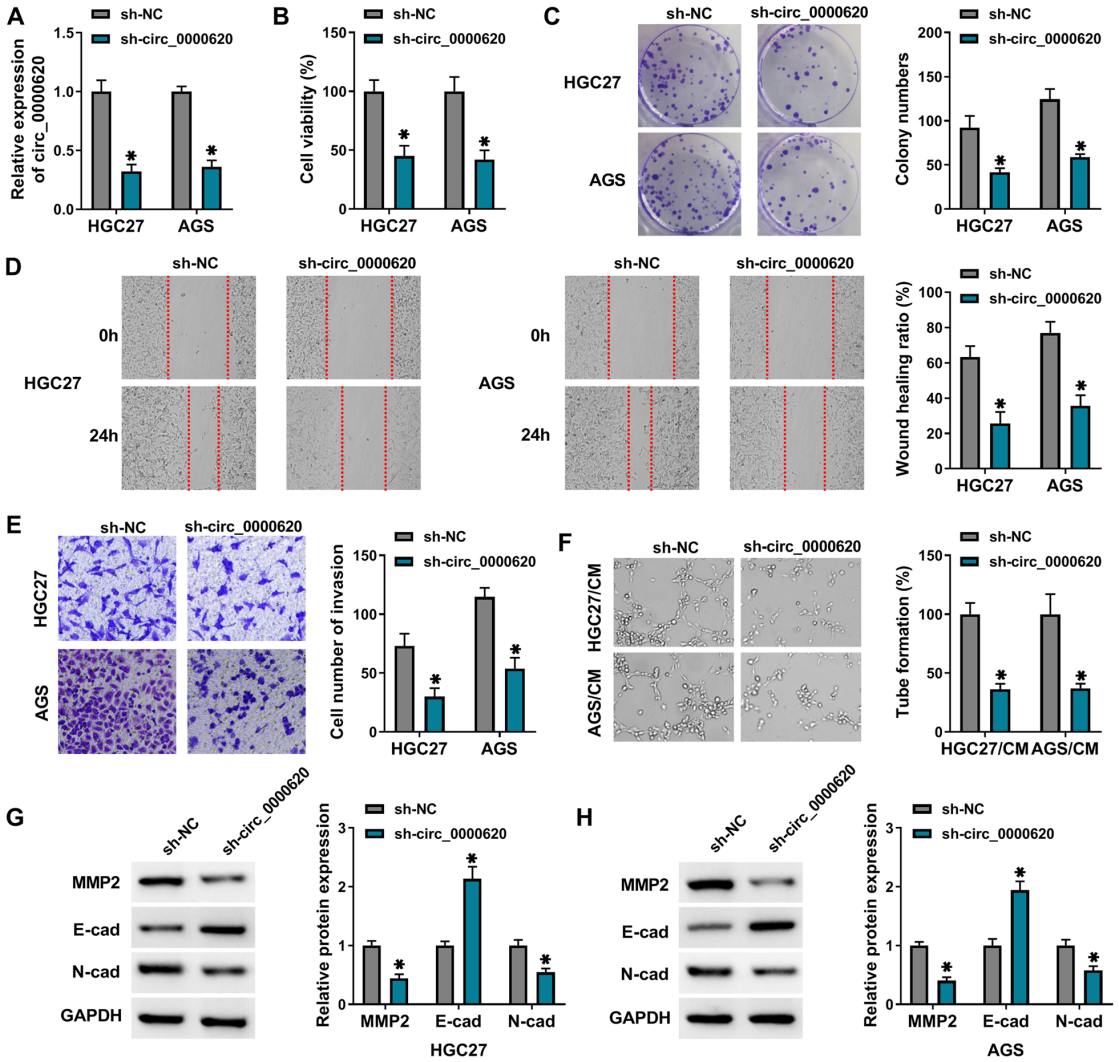
Effects of circ_0000620 knockdown on the proliferation, tube formation and invasion of GC cells. HGC27 and AGS cells were transfected with sh-NC or sh-circ_0000620 for 36 h. **A** The expression of circ_0000620 was detected by qRT-PCR. **B** Cell viability was analyzed by CCK-8 assay. **C** The ability of colony formation was tested through colony formation assay. **D** Wound healing assay was implemented to assess cell migration capacity. **E** The invasive ability was assessed by transwell invasion assay. **F** The angiogenic ability was estimated through tube formation assay. **G**, **H** The expression of metastasis-associated protein markers (MMP2, E-cad and N-cad) was analyzed by western blot assay. Three repetitions were performed in each experiment, with three parallels every time. *p < 0.05

A series of experiments were performed to investigate the biological significance of circ_0000620 in GC progression. First, qRT-PCR result showed that the level of circ_0000620 was significantly declined in HGC27 and AGS cells transfected with sh-circ_0000620 compared to sh-NC group (Fig. 2A). CCK-8 assay revealed that silence of circ_0000620 triggered the obvious reduction of cell viability of HGC27 and AGS cells (Fig. 2B). Also, colony formation ability was notably suppressed by circ_0000620 knockdown (Fig. 2C). From the data of wound healing assay and transwell assay, we monitored that cell migration (Fig. 2D) and invasion (Fig. 2E) capacities were inhibited after downregulation of circ_0000620 in HGC27 and AGS cells. Tube formation assay demonstrated that the average number of complete tubular structures formed by HUVECs was decreased in the CM from circ_0000620-silenced HGC27 and AGS cells (Fig. 2F). Western blot assay manifested that E-cad expression was increased while MMP2 and N-cad levels were reduced in HGC27 and AGS cells after circ_0000620 knockdown (Fig. 2G H). These data suggested that circ_0000620 knockdown inhibited proliferation, metastasis and angiogenesis of GC cells.

### Expression pattern of MMP2 in GC tissues and cells

**Fig. 3:**
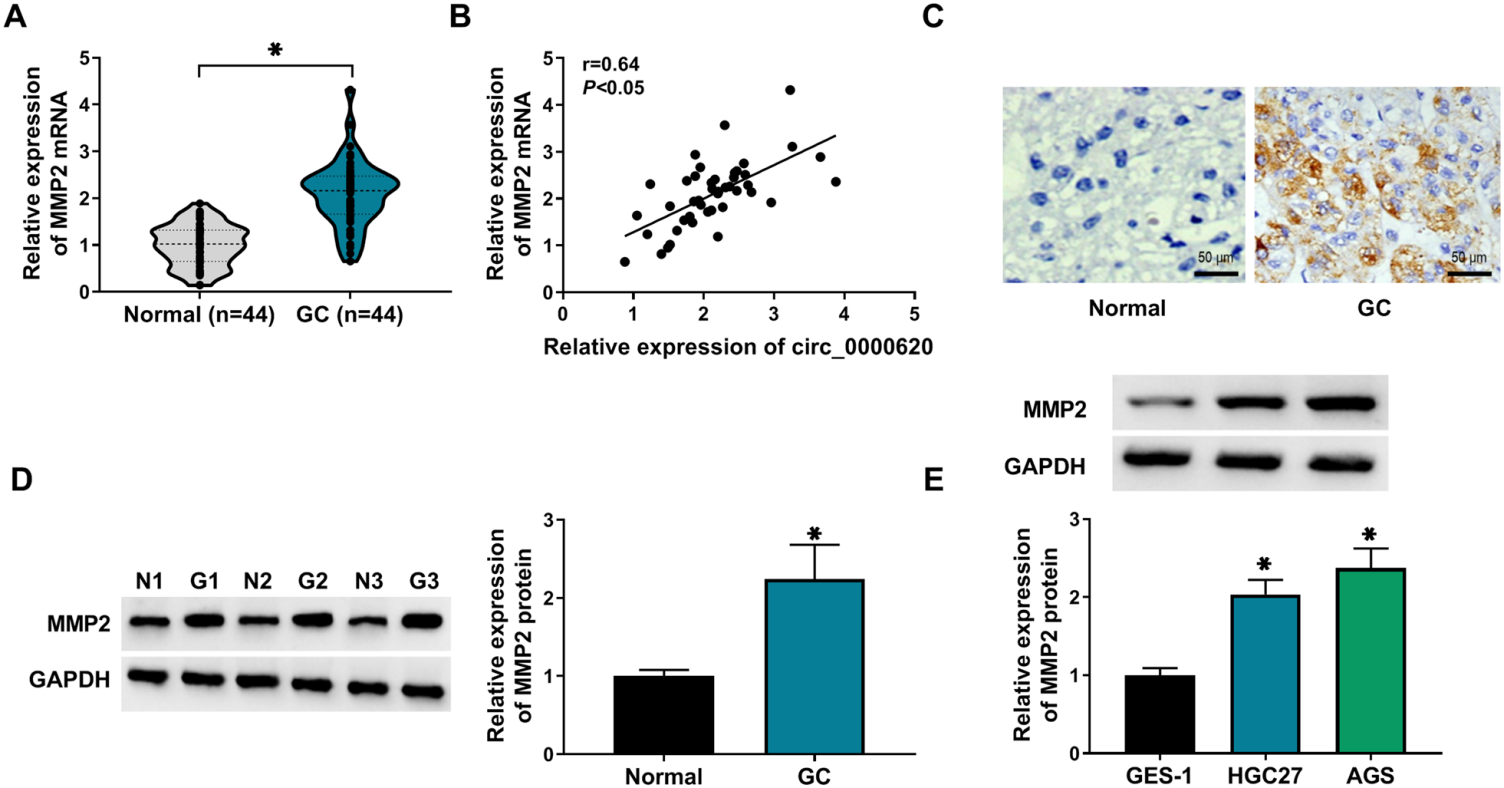
Expression pattern of MMP2 in GC tissues and cells. **A** The mRNA expression of MMP2 was determined by qRT-PCR assay in GC tissues and adjacent normal tissues. **B** The linear correlation of circ_0000620 level and MMP2 expression in 44 GC tissues was analyzed by Pearson’s correlation coefficient. **C** Representative IHC images for MMP2 expression in GC tissues and adjacent normal tissues. **D**, **E** The protein level of MMP2 was tested by western blot in GC tissues and adjacent normal tissues, normal GES-1 cells and GC cells (HGC27 and AGS). Three repetitions were performed in each experiment, with three parallels every time. *p < 0.05

The significant upregulation of MMP2 mRNA level was observed in GC tissues (n = 44) relative to the adjacent normal tissues (n = 44) (Fig. 3A). Interestingly, Pearson’s correlation analysis presented that MMP2 was positively associated with circ_0000620 in 44 cases of GC tissues (Fig. 3B). IHC and western blot assays showed that MMP2 protein level was markedly elevated in GC tissues compared with adjacent normal tissues (Fig. 3C, D). Additionally, the protein expression of MMP2 was upregulated in GC cells (HGC27 and AGS) contrasted to GES-1 cells (Fig. 3E). MMP2 was highly expressed in GC tissues and cells.

### MMP2 overexpression overturned the effects of circ_0000620 silence on the progression of GC cells

**Fig. 4:**
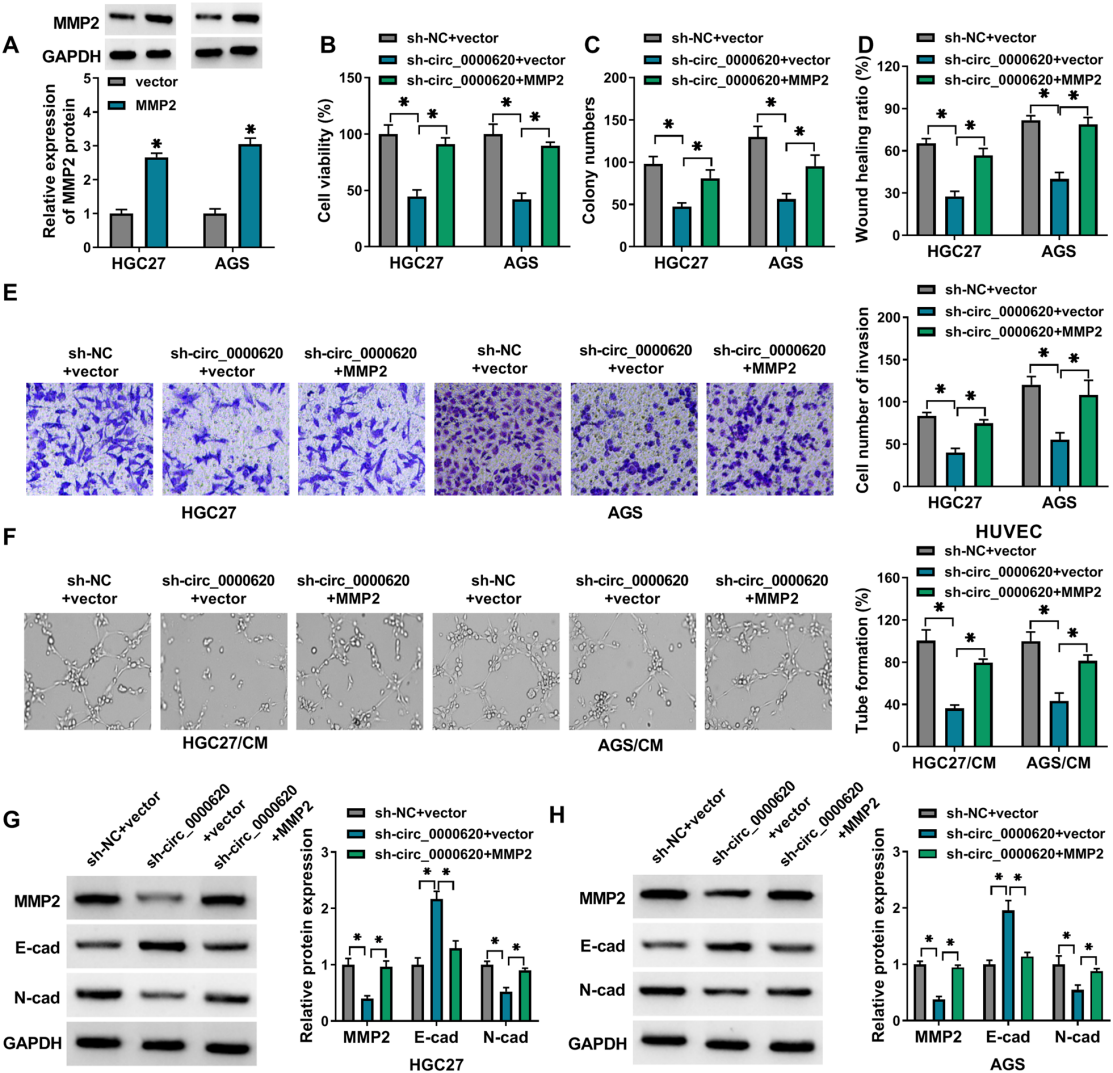
MMP2 overexpression overturned the effects of circ_0000620 silence on the progression of GC cells. **A** The expression of MMP2 in HGC27 and AGS cells transfected with vector or MMP2 was detected by western blot. **B**-**H** HGC27 and AGS cells were transfected with sh-NC + vector, sh-circ_0000620 + vector or sh-circ_0000620 + MMP2 for 36 h. CCK-8 assay, colony formation assay, wound healing assay, transwell assay and tube formation assay were conducted for the detection of cell viability (**B**), colony formation (**C**), cell migratory ability (**D**), cell invasive ability (**E**) and tube formation capacity (**F**), respectively. **G**, **H** The expression of MMP2, E-cad and N-cad was examined by western blot assay. Three repetitions were performed in each experiment, with three parallels every time. *p < 0.05

Furthermore, the association between circ_0000620 and MMP2 was explored via the rescued experiments. The protein level of MMP2 was remarkably upregulated in HGC27 and AGS cells transfected with MMP2 vector, relative to control group (Fig. 4A). MMP2 overexpression distinctly reversed the inhibiting actions of circ_0000620 knockdown on cell viability (Fig. 4B), colony formation (Fig. 4C), migration and invasion capacities (Fig. 4D, E) and tube formation ability (Fig. 4F). Additionally, the sh-circ_0000620-induced E-cad upregulation but MMP2 and N-cad downregulation were alleviated by the introduction of MMP2 in HGC27 and AGS cells (Fig. 4G, H). These data suggested that the inhibiting effects of circ_0000620 knockdown on the malignant behaviors of GC cells were achieved by downregulating the MMP2 expression.

### Circ_0000620 served as a molecular sponge of miR-671-5p to regulate MMP2 expression

**Fig. 5:**
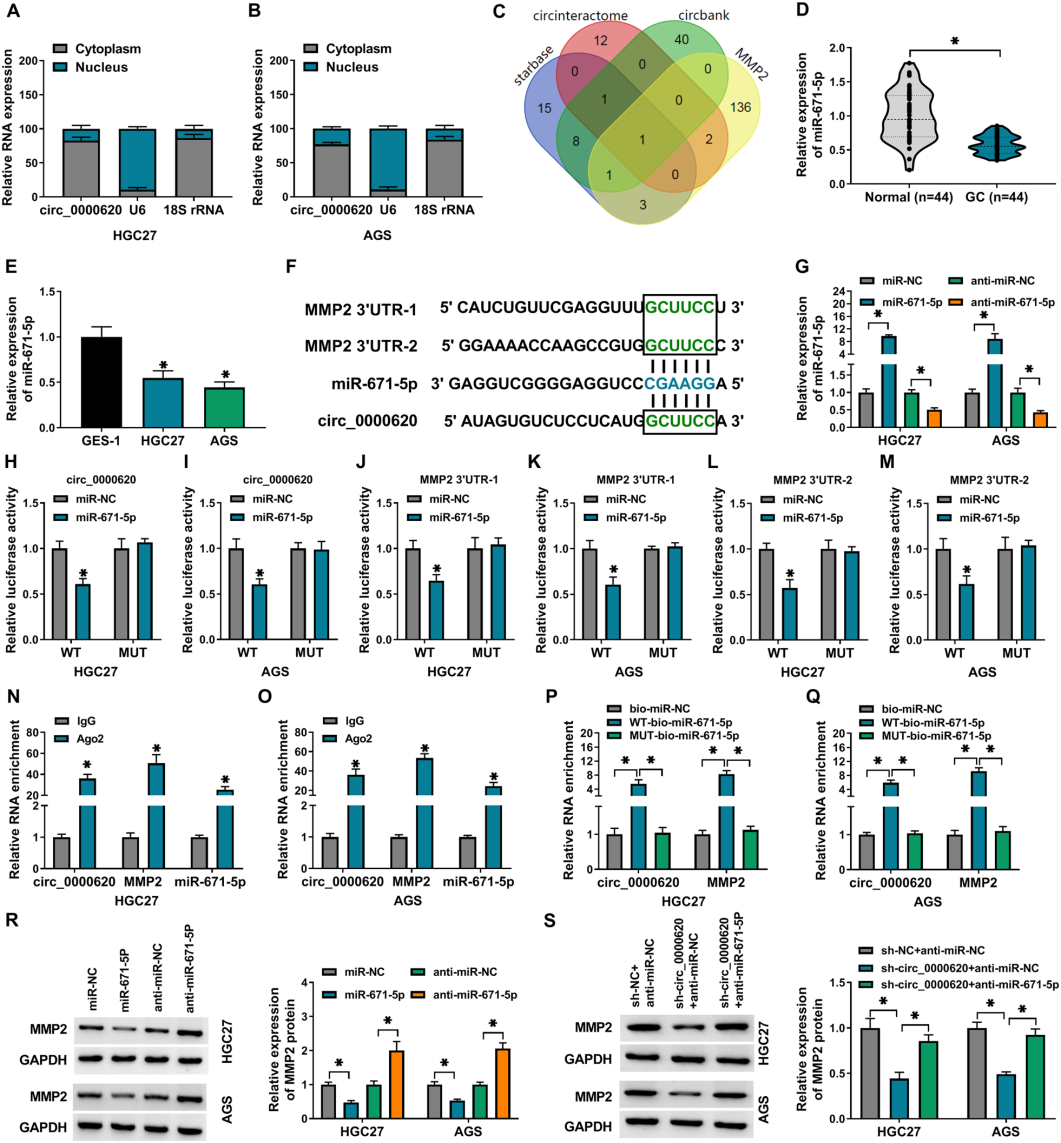
Circ_0000620 served as a molecular sponge of miR-671-5p to regulate MMP2 expression. **A**, **B** The subcellular localization of circ_0000620 was analyzed in GC cells using subcellular fractionation assays, with 18 S rRNA and U6 as internal controls. **C** Venn diagram was performed to analyze the potential miRNAs that bind to circ_0000620 and MMP2. The level of miR-671-5p was tested by qRT-PCR assay in GC tissues and adjacent normal tissues (**D**), as well as in normal GES-1 cells and GC cells (HGC27 and AGS) (**E**). **F** The complementary sites between miR-671-5p and circ_0000620 or MMP2 (MMP2 3΄UTR-1 and MMP2 3΄UTR-2). **G** The expression of miR-671-5p was detected by qRT-PCR in HGC27 and AGS cells transfected with miR-671-5p mimic, anti-miR-671-5p or corresponding contrasts. **H**–**M** Relative luciferase activities in HGC27 and AGS cells co-transfected with circ_0000620 or MMP2 reporters and miR-NC or miR-671-5p were tested through luciferase reporter assay. **N**–**Q** RIP assay (**N**, **O**) and RNA pull-down assay (**P**, **Q**) were used to confirm the interaction between circ_0000620 or MMP2 and miR-671-5p. **R** and **S** The protein level of MMP2 was determined by western blot assay after transfection with miR-671-5p mimic, anti-miR-671-5p, sh-circ_0000620 + anti-miR-671-5p or corresponding contrasts. Three repetitions were performed in each experiment, with three parallels every time. *p < 0.05

The subcellular fractionation assay showed that circ_0000620 was mainly localized in the cytoplasm of HGC27 and AGS cells (Fig. 5A, B). To explore the potential molecular mechanism of circ_0000620 and MMP2 in GC, bioinformatic analysis was performed to analyze the potential target miRNAs that potentially bind to circ_0000620 and MMP2. Circinteractome, starBase and circBank were used to predict the target miRNAs of circ_0000620, and starBase software was applied to predict the miRNAs that had complementary sites with MMP2 3΄UTR. Venn diagram displayed that only miR-671-5p contained the binding regions with circ_0000620 and MMP2 (Fig. 5C). The qRT-PCR assay showed that miR-671-5p level was significantly reduced in GC tissues (n = 44) and HGC27/AGS cells relative to adjacent normal tissues (n = 44) and normal GES-1 cells (Fig. 5D, E). The putative complementary sites between miR-671-5p and circ_0000620 or MMP2 (MMP2 3΄UTR-1 and MMP2 3΄UTR-2) were shown in Fig. 5F. The miR-671-5p level was increased following the transfection of miR-671-5p mimic, but miR-671-5p was downregulated after anti-miR-671-5p transfection (Fig. 5G). Dual-luciferase reporter assay demonstrated that overexpression of miR-671-5p reduced luciferase activity of circ_0000620 WT, MMP2 3΄UTR-1 WT and MMP2 3΄UTR-2 WT reporters, but it had no much influence on luciferase activity of MUT plasmids in HGC27 and AGS cells (Fig. 5H-M). RIP assay results suggested that circ_0000620, miR-671-5p and MMP2 levels were higher in Ago2 group than these in IgG group (Fig. 5N, O). Furthermore, RNA pull-down assay revealed that circ_0000620 and MMP2 were captured by WT-bio-miR-671-5p but not of MUT-bio-miR-671-5p or bio-miR-NC in HGC27 and AGS cells (Fig. 5P and Q). Thus, circ_0000620 or MMP2 could interact with miR-671-5p in GC cells. Moreover, western blot exhibited that MMP2 expression was downregulated by miR-671-5p overexpression while it was upregulated following miR-671-5p silence in HGC27 and AGS cells (Fig. 5R). The circ_0000620 knockdown inhibited the expression of MMP2, and the introduction of anti-miR-671-5p recovered this effect (Fig. 5S). In summary, circ_0000620 promoted MMP2 expression by directly interacting with miR-671-5p in GC cells.

### MiR-671-5p inhibitor reversed the inhibitory effects of circ_0000620 knockdown on GC cell progression

**Fig. 6:**
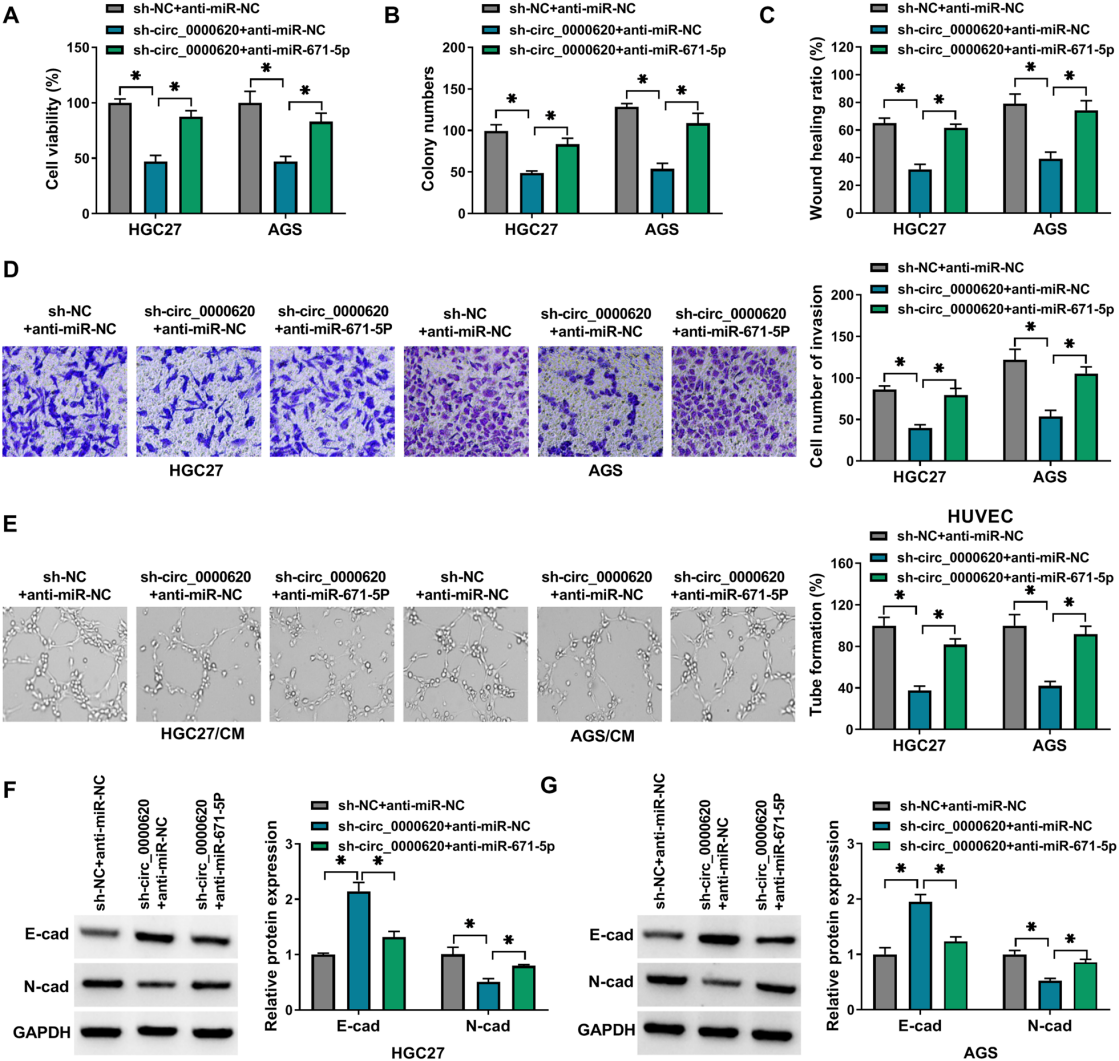
MiR-671-5p inhibitor reversed the inhibitory effects of circ_0000620 knockdown on GC cell progression. HGC27 and AGS cells were transfected with sh-NC + anti-miR-NC, sh-circ_0000620 + anti-miR-NC or sh-circ_0000620 + anti-miR-671-5p. Cell viability (**A**), colony formation (**B**), cell migratory ability (**C**), cell invasive ability (**D**) and tube formation capacity (**E**) were respectively tested by CCK-8 assay, colony formation assay, wound healing assay, transwell assay and tube formation assay. **F**, **G** The expression of E-cad and N-cad was detected by western blot assay. Three repetitions were performed in each experiment, with three parallels every time. *p < 0.05

The regulatory relation between circ_0000620 and miR-671-5p was further researched through the transfection of sh-circ_0000620, sh-circ_0000620+anti-miR-671-5p or the matched control groups. Functional assays revealed that circ_0000620 knockdown-induced inhibitory effects on cell viability (Fig. 6A), colony formation (Fig. 6B), migration/invasion (Fig. 6C, D) and tube formation ability (Fig. 6E) were partly abolished by the downregulation of miR-671-5p. Furthermore, the effects of si-circ_0000620 on protein levels of E-cad and N-cad were remarkably reversed by anti-miR-671-5p (Fig. 6F, G). All in all, circ_0000620 inhibited GC cell progression by targeting miR-671-5p.

### Knockdown of circ_0000620 inhibited GC tumor growth in vivo *undefined*

**Fig. 7:**
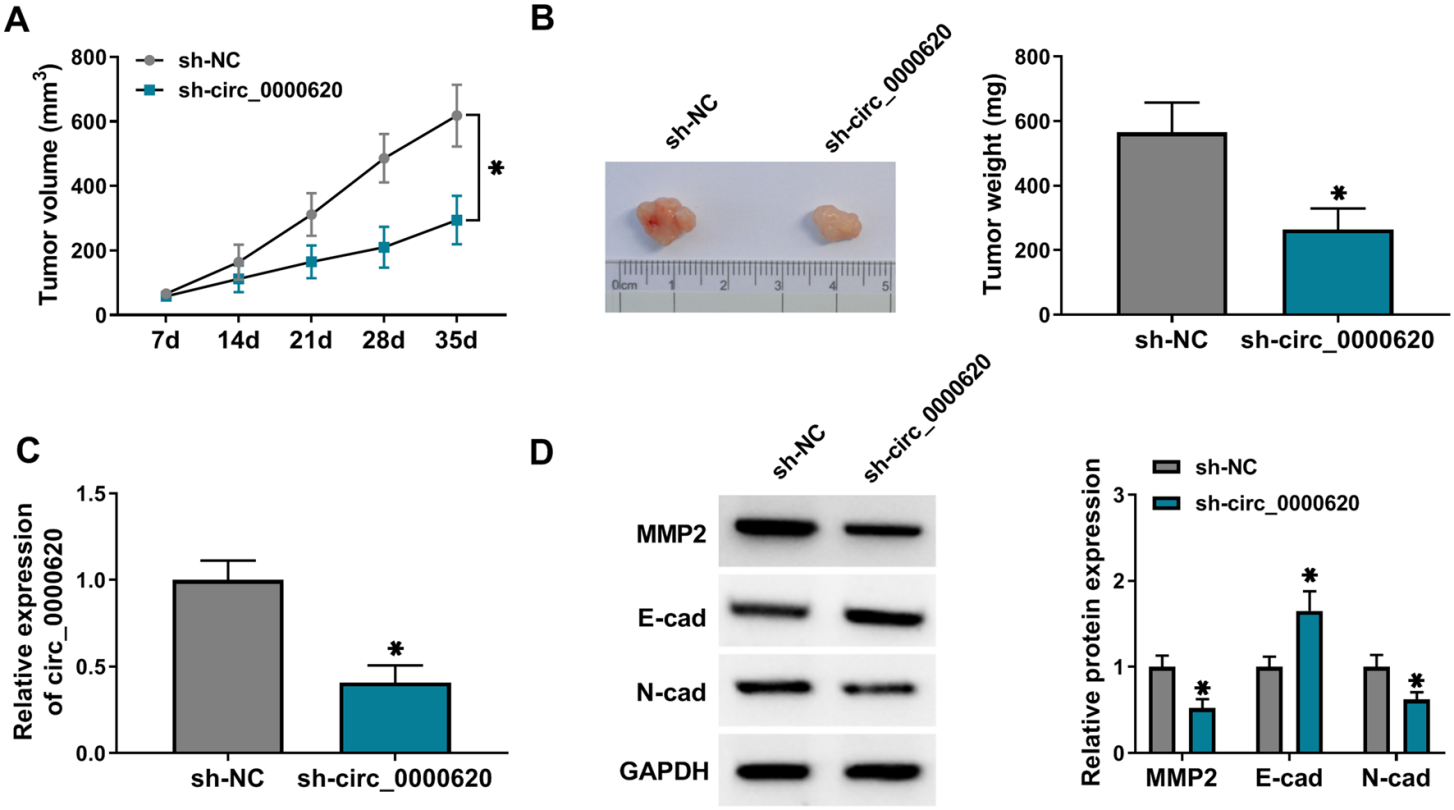
Knockdown of circ_0000620 inhibited tumor growth in vivo. HGC27 cells transfected with sh-NC or sh-circ_0000620 were injected into nude mice. **A** Tumor volume was measured every 7 days. **B** Tumor weight was measured after injection for 35 days. **C** The expression of circ_0000620 was detected by qRT-PCR in mice tumor tissues. **D** The protein levels of MMP2, E-cad and N-cad were detected by western blot. Three repetitions were performed in each experiment, with three parallels every time. *p < 0.05

To explore the role of circ_0000620 in GC in vivo, HGC27 cells with transfection of sh-NC or sh-circ_0000620 were injected into the nude mice. Tumor volume and weight were evidently suppressed in sh-circ_0000620 group, compared with sh-NC group (Fig. 7A, B). The qRT-PCR revealed that circ_0000620 was downregulated in sh-circ_0000620 group relative to sh-NC group (Fig. 7C). With the downregulation of circ_0000620, we found that E-cad protein level was increased but the levels of MMP2 and N-cad were reduced in tumor tissues (Fig. 7D). These results suggested that circ_0000620 knockdown inhibited tumor growth in vivo.

## Discussion

Increasing evidence demonstrated that circRNAs could function as key players in GC pathogenesis, which might provide a novel field of diagnostic and therapeutic opportunities for GC patients [16]. In this study, we explored the function of circ_0000620 and first identified the circ_0000620/miR-671-5p/MMP2 signal axis in GC malignant progression.

CircRNAs have important regulation in pathological behaviors of various cancers, which is partly achieved by acting as “miRNA sponges” [15, 32]. Many circRNAs are shown to be differentially expressed and play important roles in carcinogenesis of GC [16]. For instance, circ_0004104 accelerated the progression of GC via the miR-539-3p/RNF2 axis [33]. However, the role of circ_0000620 in the progression of GC is fully unclear. Niu et al. indicated that circ_0000620 was highly expressed in GC cells [19]. In accordance with the result, we found that circ_0000620 expression was upregulated in GC tissues and cells. The loss-of-function experiments demonstrated that circ_0000620 downregulation blocked GC cell proliferation, migration, invasion and angiogenesis. Besides, animal experiments also affirmed that circ_0000620 could facilitate GC tumor growth *in vivo*. Thereby, circ_0000620 acted as a carcinogenic role in GC. Knockdown of circ_0000620 might be used for anti-metastasis and anti-angiogenesis to impede the malignancy of GC patients.

MMP2 have been identified as an essential factor in the switch to the angiogenic phenotype in some tumors such as chondrosarcoma [34] and glioma [35]. MMP2 expression was associated with tumor size, invasion and metastasis, microvessel density and VEGF expression in GC [31, 36]. Moreover, Chen et al. further found that MMP2 could promote tumor angiogenesis in GC [30]. Consistent with the previous report [30], our data showed that MMP2 expression was markedly upregulated in GC tissues and cells. More interestingly, MMP2 overexpression mitigated the effects of circ_0000620 silence on the progression of GC cells. Therefore, MMP2 was partly responsible for circ_0000620-mediated GC development.

The circRNA/miRNA/mRNA regulatory network has been reported in various diseases, including GC [37, 38]. Herein, we discovered that circ_0000620 could regulate the expression of MMP2 through targeting miR-671-5p. MiR-671-5p has been identified as a crucial player in cancer regulation. For instance, Li et al. demonstrated that miR-671-5p impeded the progression of esophageal squamous cell carcinoma (ESCC) by downregulating FGFR2 [29]. Xin et al. elucidated that miR-671-5p inhibited tumor proliferation and cell cycle progression in osteosarcoma [39]. Besides, miR-671-5p has also been reported to be involved in GC development [25, 40]. This study validated the downregulation of miR-671-5p in GC tissues and cells, which was in line with the previous reports [25, 40]. Moreover, our data manifested that the regulatory effects of circ_0000620 were ascribed to serve as a miR-671-5p sponge in GC cells.

This study still has some limitations. Firstly, some experiments such as in situ assay cannot be performed because of the limited condition and fund. Secondly, the circ_0000620/miR-671-5p/MMP2 axis in vivo needs further identification. Thirdly, whether other miRNA/mRNA networks are related to the oncogenic function of circ_0000620 remains to be explored. Last but not the least, the downstream signaling pathways of MMP9 are unclear. It is interesting to discover the signaling pathway underlying the circ_0000620/miR-671-5p/MMP2 axis, which may contribute to understanding the functional mechanism of circ_0000620 in GC. In addition, the potential of circ_000620 as a diagnostic or therapeutic biomarker will be validated in future.

**Fig. 8:**
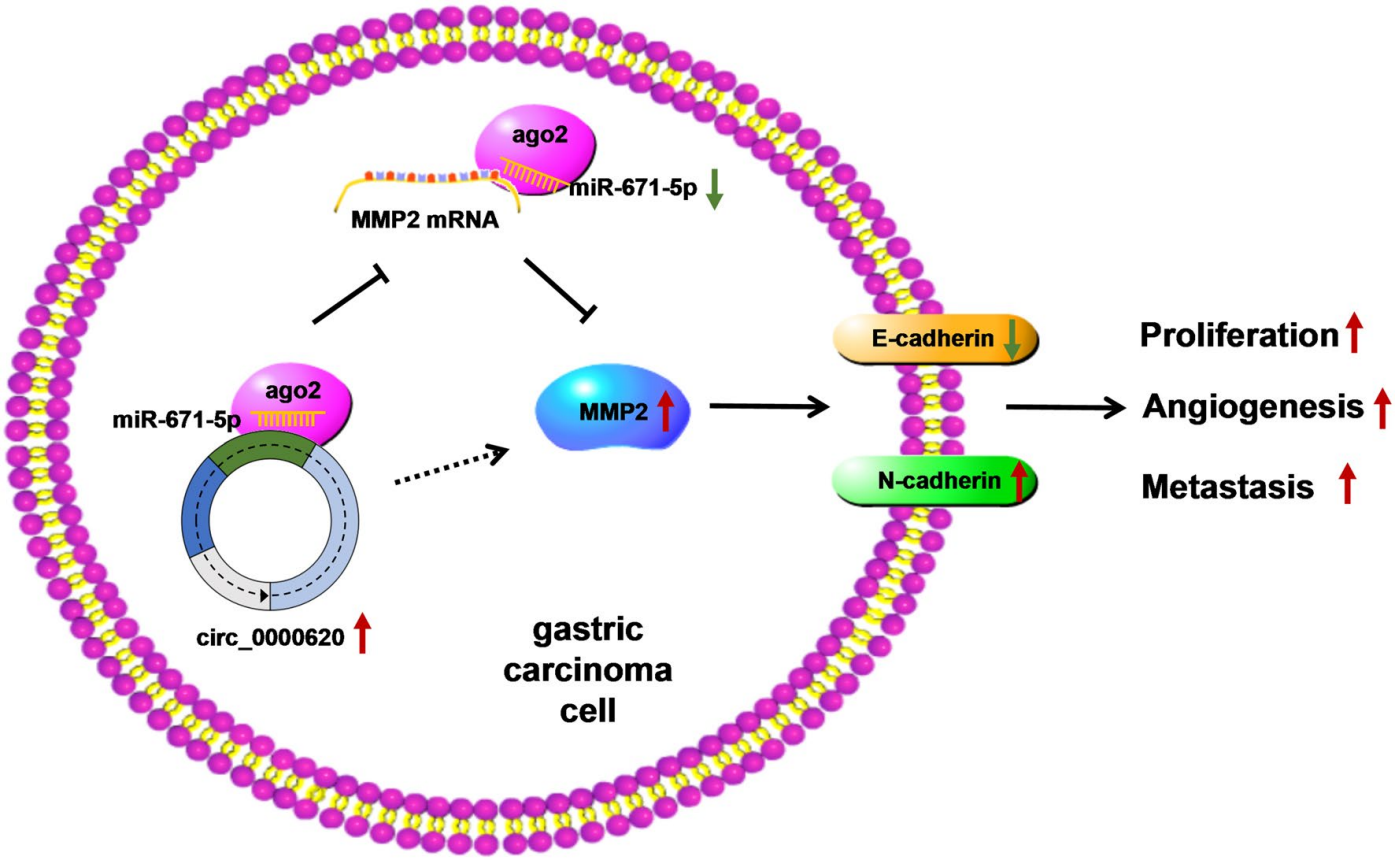
Schematic diagram of working mechanism behind the oncogenic role of circ_0000620 in GC cells. Circ_0000620 competitively combined with miR-671-5p to increase the MMP2 expression, thereby promoting GC cell proliferation, metastasis and angiogenesis. Additional file 1; Fig. S1. Circ_0000620 was the most significantly upregulated in 5 GC samples. The levels of circ_0000620, circ_0000847 and circ_0000567 were detected by qRT-PCR in 5 pairs of GC and normal tissues. Three repetitions were performed in the qRT-PCR experiment, with three parallels every time. *p < 0.05

Taken together, this study suggested that circ_0000620 facilitated GC cell proliferation, metastasis and angiogenesis via upregulating MMP2 expression through sponging miR-671-5p (Fig. 8). Our research unraveled the functional role and regulatory mechanism of circ_0000620 in GC.

## Supplementary Information

**Additional file 1: Fig. S1. **Circ_0000620 was the most significantly upregulated in 5 GC samples. The levels of circ_0000620, circ_0000847 and circ_0000567 were detected by qRT-PCR in 5 pairs of GC and normal tissues. Three repetitions were performed in the qRT-PCR experiment, with three parallels every time. *p < 0.05.

## Data Availability

The analyzed data sets generated during the present study are available from the corresponding author on reasonable request
