# Supplementary figures and images for: Circ_0000620 acts as an oncogenic factor in gastric cancer through regulating MMP2 expression via sponging miR-671-5p
Source: J Biol Res (Thessalon). 2021 Dec 31;28:23. doi: 10.1186/s40709-021-00154-5 (PMC8720221; doi:10.1186/s40709-021-00154-5)

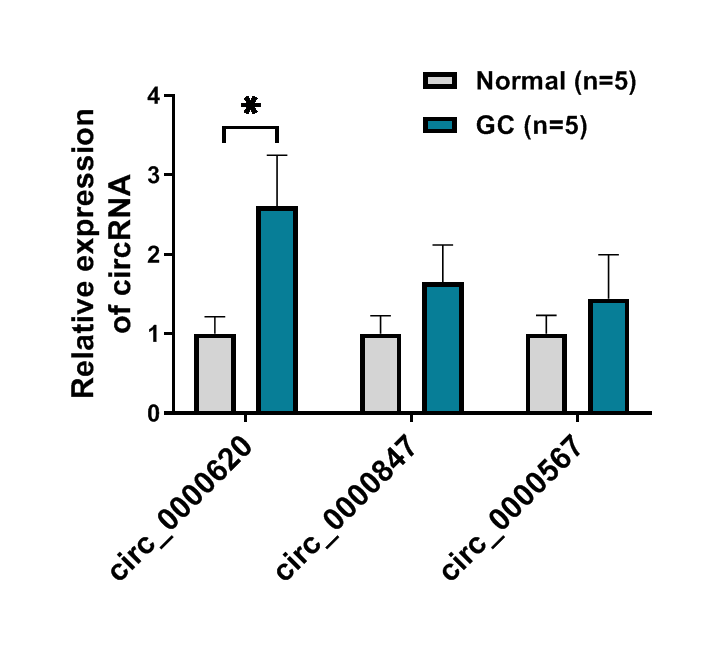

Supplement: Supplementary file 1 — Additional file 1: Fig. S1. Circ_0000620 was the most significantly upregulated in 5 GC samples. The levels of circ_0000620, circ_0000847 and circ_0000567 were detected by qRT-PCR in 5 pairs of GC and normal tissues. Three repetitions were performed in the qRT-PCR experiment, with three parallels every time. *p < 0.05. [file 40709_2021_154_MOESM1_ESM.tif]
